# Leukemia inhibitory factor increases the proliferation of human endometrial stromal cells and expression of genes related to pluripotency

**Published:** 2017-04-10

**Authors:** Mojdeh Salehnia, Mehri Fayazi, Shokreya Ehsani

**Affiliations:** 1 *Anatomy Department, Faculty of Medical Sciences, Tarbiat Modares University, Tehran, Iran.*; 2 *Department of Medical Sciences, Najafabad Branch, Islamic Azad University, Najafabad, Iran.*

**Keywords:** Endometrium, Cell proliferation, Gene expression, Leukemia inhibitory factor, Stromal cells

## Abstract

**Background:**

Concerning the low population of human endometrial mesenchymal cells within the tissue and their potential application in the clinic and tissue engineering, some researches have been focused on their in vitro expansion.

**Objective:**

The aim of this study was to evaluate the effect of leukemia inhibitory factor (LIF) as a proliferative factor on the expansion and proliferation of human endometrial stromal cells.

**Materials and Methods:**

In this experimental study, the isolated and cultured human endometrial stromal cells from women at ovulatory phase aged 20-35 years, after fourth passage were divided into control and LIF-treated groups. In the experimental group, the endometrial cells were treated by 10 ng/ml LIF in culture media and the cultured cells without adding LIF considered as control group. Both groups were evaluated and compared for proliferation rate using MTT assay, for CD90 marker by flow cytometric analysis and for the expression of Oct4, Nanog, PCNA and LIFr genes using real-time RT-PCR.

**Results:**

The proliferation rate of control and LIF-treated groups were 1.17±0.17 and 1.61±0.06 respectively and there was a significant increase in endometrial stromal cell proliferation following in vitro treatment by LIF compared to control group (p=0.049). The rate of CD90 positive cells was significantly increased in LIF-treated group (98.96±0.37%) compared to control group (94.26±0.08%) (p=0.0498). Also, the expression ratio of all studied genes was significantly increased in the LIF-treated group compared to control group (p=0.0479).

**Conclusion:**

The present study showed that LIF has a great impact on proliferation, survival, and maintenance of pluripotency of human endometrial stromal cells and it could be applicable in cell therapies.

## Introduction

The human endometrium is a highly dynamic tissue that undergoes 400 menstrual cycles during a woman’s life (1). This level of tissue regeneration is related to stem cells in the endometrium. For the first time, Chan et al in 2004 identified and characterized stem cells in endometrial tissue (2). Characterization of endometrial stromal cells revealed mesenchymal stem cell properties such as adherence to plastic, clonogenicity, expression of CD90, CD73 and CD105 markers and differentiation to adipogenic, osteogenic and neurogenic fates in vitro (3-8). Leukemia inhibitory factor (LIF) belongs to the family of interleukin-6 cytokines (9). LIF interacts with cells through the gp130 and LIF receptor (LIFR) upon binding to receptor subunits, and this dimerization results in the activation of associated Jak tyrosine kinases and initiation of intracellular signaling pathways (9-12). The LIF acts an anti-apoptotic and a proliferative factor and also inhibits differentiation of stem cells (13-16). 

It is recognized that endometrial stem cells may be a valuable therapeutic tool in the regenerative medicine field (1). So, expansion of human endometrial stem cells has become a focus for some researchers, concerning the valuable application of human endometrial stem cells in clinic and tissue engineering. The ability of the cytokine LIF to maintain the pluripotency of embryonic stem cells and other types of stem cell has previously shown (9, 17-21). Some investigations reported that MSC self-renewal and undifferentiation could be sustained in culture media supplemented with LIF (10, 13).

However, there are some reports for characterization of endometrial stem cells but the possible effect of LIF on the proliferative expansion of these cells remains elusive. In this study, we were attempting to evaluate the proliferation effect of LIF on the expansion of human endometrial stem cells.

## Materials and methods

For this experimental study human endometrial samples were collected from women at ovulatory phase aged 20-35 years (n=15) undergoing hysteroscopy for non-endometrial pathologic conditions in Emam Khomeini Hospital (Tehran, Iran). They had not taken exogenous hormones for three months prior to hysteroscopy. The normality of endometrial tissue was approved from pathology reports evaluated by experienced histopathologists, according to well-established histological criteria for the normal menstrual cycle. 

**Experimental design**

The human endometrial cells were isolated mechanically and enzymatically, and then cultured to the fourth passage. Then isolated human endometrial cells were divided into two groups including control and LIF-treated (experimental) groups. In the LIF-treated group, the cells were treated with 10 ng/ml LIF and the cultured cells without LIF were considered as control group. Finally, the proliferation rate of endometrial cells in both groups was evaluated and compared using MTT assay, flow cytometric and real-time RT-PCR analysis.

**Preparations and in vitro culture of human endometrial cells**

Preparation of human endometrial cells was done according to the method described earlier by Chan *et al* (2). Briefly, human endometrial tissues were collected and washed in phosphate buffer saline (PBS). Then the tissues were minced in a medium containing Dulbecco Modified Eagle Medium/Hams F-12 (DMEM/F-12), antibiotics and 10% fetal bovine serum (FBS; Invitrogen, Manchester, UK). Then, human endometrial tissues were cut into single cell suspensions using collagenase type 3 (300 µg/ml), deoxyribonuclease type I (40 µg/ml), and mechanical methods for 50-60 min. Then they were pipetted and filtered using meshes of 150, 100, 40 sieve (BD Biosciences, Erembodegem, Belgium) respectively to dissociate single cells from epithelial cells, debris and undigested tissue fragments (22). 

The isolated endometrial stromal cells were seeded at a concentration of 3×10^5^ cells into 6 well chamber slides using complete media and then incubated at 37^o^C in 5% CO_2_. The media was changed every 3 days. The cells were passaged and sub-cultured when the cultures reached 80% of confluency. The isolated human endometrial cells at fourth passage were divided into two groups including control and LIF-treated groups. In the LIF-treated group, the cells were treated by 10 ng/ml LIF and the cultured cells without LIF considered as control group. They were taken for the following assessments.

**Cell proliferation of LIF treated cells by MTT assay**

Cellular expansion growth rate was evaluated by MTT assay. For evaluation of endometrial cells proliferation following LIF, the tetrazolium compound MTT [3-(4,5-dimethylthiazol-2-yl) -2,5-diphenyltetrazolium bromide] was added to cultured cells at fourth passage. Briefly, cells were seeded into two different groups of wells at a density of 2×10^4^ cells/in 24 well, and then volumes of 100 μl of fresh medium and supplemented serum were dispensed in triplicates with 0 and 10 ng/ml of LIF. Then two groups of wells were incubated at 37^o^C in 5% CO_2_ for 24 hr and 72 hr respectively. At 24 hr and 72 hr of the culture, 100 μL of 0.5 mg/ml MTT reagent was added to each well and the cells were incubated for 4 hr at 37^o^C. 

At the end of incubation period, MTT was reduced by metabolically active cells, so insoluble purple formazan dye crystals deposits produced within the cells. After removing of MTT solution, 100 μL of dimethyl sulfoxide (DMSO) were added to each well that causes destructing cell membranes and then the absorbance was read using a spectrophotometer. The rate of tetrazolium reduction is directly proportional to the rate of cell proliferation (23). Finally, the optical density (OD) was measured at 570 nm using a microplate reader (Bio-Rad, California, USA). The average values from triplicate readings of LIF-treated versus un-treated wells were determined and used to calculate the stimulation index (SI) as follow: SI = mean of OD values of mitogen LIF-treated wells/mean of OD values of un-treated wells (24).

**Flow cytometry of human cultured endometrial stromal cells**

The immunophenotype of isolated endometrial stromal cells of two groups including control and LIF-treated were assessed using flow cytometric analysis for CD90 as a mesenchymal stem cell marker (n=4). Cultured endometrial cells were trypsinised and resuspended 5×10^5^ cells in 100 μl of PBS. The cells were incubated with the direct APC-conjugated antibody (CD90) (1:100 dilutions) at 4^o^C for 1 hr, they were washed twice with PBS. The labeled cells were put in 100 μl of PBS and examined with a FACS Calibur apparatus (Becton Dickinson). 

**Real-time RT-PCR assay**

The expression of Oct4, Nanog, PCNA and LIFr genes was evaluated in endometrial cells of control and LIF-treated (n=3). The primers were designed using Perl Primer software (3.05) (**Table 1**). Designed primers were ordered and synthesized at CinnaGen Co. (Tehran, Iran). The housekeeping gene, GAPDH, was used as an internal control. The PCR reactions were carried out by SYBR Green Master Mix method (Applied Biosystems, UK). The mRNA expression of Oct4, Nanog, PCNA and LIFr genes in two groups was quantified using the ABI 7500 Sequence Detector (Applied Biosystems, UK) according to the manufacturer's instructions. The PCR protocol included an initial denaturation at 95^o^C for 5 min; followed by 40 cycles consisting of denaturation at 95^o^C for 15 sec. Annealing was carried out, then extension at 72^o^C for 30 sec. 

At the end of amplification cycles, melting temperature analysis was done at 95^o^C for 15 sec, 60^o^C for 1 min and 95^o^C for 15 sec. The experiments were done in three replicates for each sample and non- template samples were considered as negative controls. For target sequence amplifications, 500 ng RNA was used per 20 μL reaction volume. After completing the PCR run, melt curve analysis was used to confirm the amplified product. The real-time PCR products were further verified by 1.5% agarose gel electrophoresis. Then relative quantization of target genes was determined using the Pfaffl method (25).

**Ethical consideration**

Ethics approval was obtained from the ethics committee of the medical faculty of Tarbiat Modares University Informed agreement was achieved from each woman.

**Statistical analysis**

Statistical analysis was done using SPSS software (version 17.0, USA). All data were presented as mean±standard errors of the means (SEM). The T-test used to detect the statistical significance between two groups. Statistical significance was identified as p<0.05.

## Results

**Morphological observation of human cultured endometrial cells**

After 24 hr, the endometrial cells adhered to the culture dish and they were rapidly expanded. The cultured cells at the end of fourth passage had spindle shape cells. Cells usually appeared elongated and spindle-shape with round nuclei. 

**Effect of LIF on cell proliferation by MTT assay**

**Figure 1:**
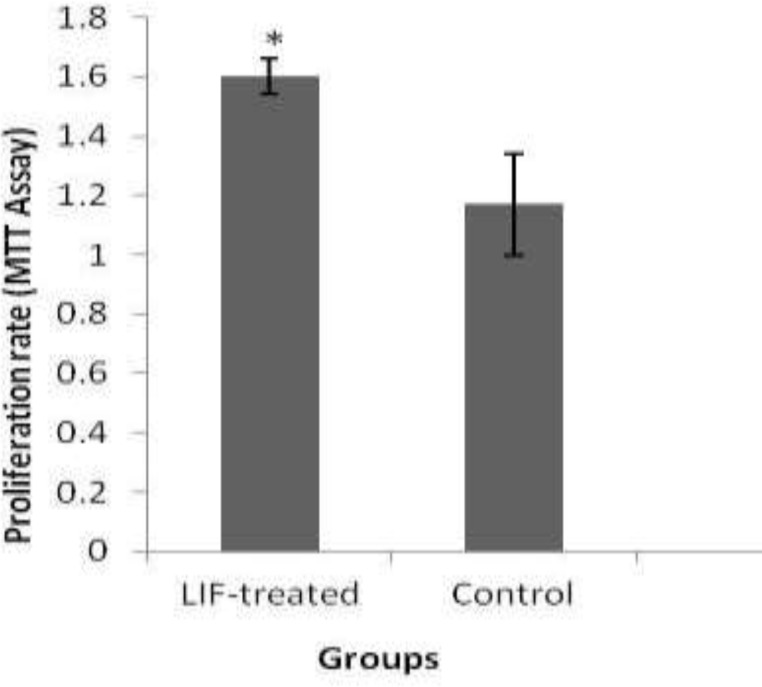
The effect of LIF on human endometrial stromal cells proliferation. *: there were significant between control and LIF-treated groups (p = 0.049

The proliferation rate of endometrial stromal cells in control and LIF-treated groups were 1.17±0.17 and 1.61±0.06 respectively. There was a significant increase in endometrial stromal cell proliferation following in vitro treatment by 10 ng/ml than untreated groups (p=0.049; Figure 1). 

**Flow cytometric analysis**

**Figure 2:**
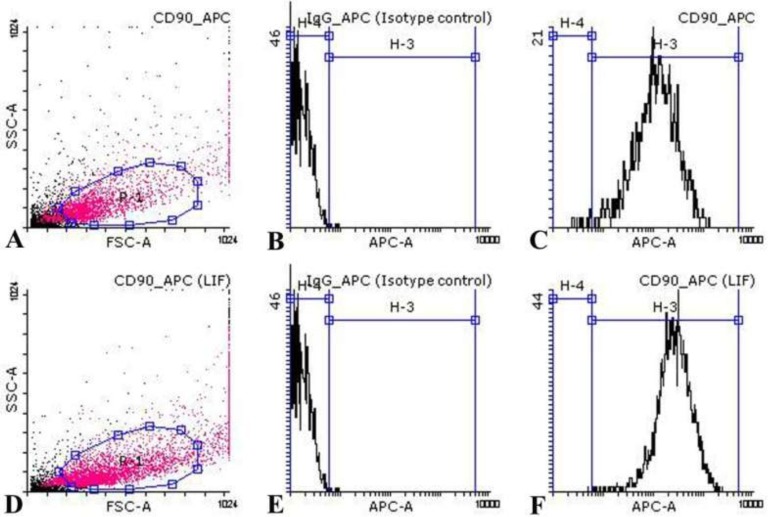
Flow cytometric analysis of cultured endometrial stromal cells in control and LIF-treated groups. Diagrams of CD90 marker of endometrial stromal cell suspensions of control (A-C) and LIF-treated (D-F) groups were shown. Analysis showed that the cultured endometrial cells are negatively stained with CD34 (D), CD31 (E), and CD9 (F). Diagrams of first (A, D), second (B, E) and third (C, F) columns are related to isotype control and test samples respectively. Each diagram is representative of four independent experiments

The proportion of CD90 positive cells in cultured endometrial stromal cells after fourth passage in control and LIF-treated groups were 94.26±0.08% and 98.96±0.37% respectively in four repeats (Figure 2). Data analysis showed that there was a significant difference between control and LIF-treated groups (p=0.0498).

**Real-time RT-PCR analysis**

**Figure 3:**
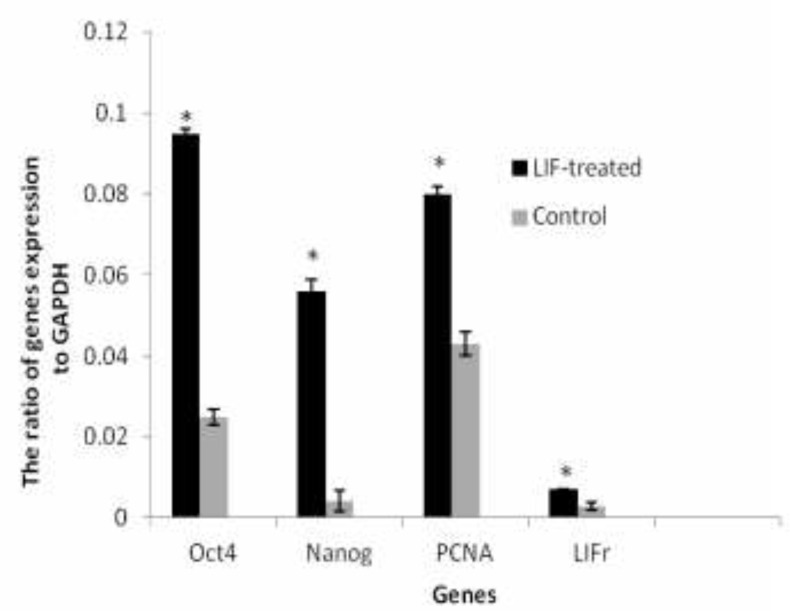
The ratio of Oct4, Nanog, PCNA and LIFr to GAPDH gene expression of endometrial cells in control and LIF-treated groups. Values are means ± SE. (*) indicates differences with control groups (P=0.0479). Each diagram is representative of three independent experiments

The level of endometrial cells Oct4, Nanog, PCNA and LIFr mRNA for GAPDH gene in control group were 0.025±0.002, 0.0042±0.0027, 0.043±0.003 and 0.003±0.001 respectively. The expression of Oct4, Nanog, PCNA and LIFr genes in endometrial cells of LIF-treated groups were 0.095±0.001, 0.056±0.003, 0.08±0.002 and 0.007±0.0001 respectively. There was a statistically significant difference between the ratio expression of these genes to housekeeping gene between control and LIF-treated groups. These levels in LIF-treated groups were significantly higher than the control groups (p=0.0479, Figure 3). GAPDH was expressed in each sample as an internal housekeeping gene, and there was no band for the non-template control (NTC) samples.

## Discussion

Results obtained in the present study for the first attempt revealed that human isolated endometrial cells which were treated LIF exhibited a significant increase in proliferative response in comparison with control groups. According to our results, LIF has a major influence on human endometrial stem cell proliferation in the culture medium. 

Many of previous studies have reported similar observations to our results. Some investigations revealed that LIF is necessary for cell survival and proliferation of progenitor/stem cells (26-30). Carter *et al* reported that LIF maintained retinal stem cell proliferation and undifferentiation, Nikolova *et al* and Mirzapour *et al* found that LIF increased proliferation of spermatogonial stem cells, Bauer et al evaluated the effect of LIF on the proliferation of neural stem cells and Furue *et al* showed proliferation and pluripotency of murine embryonic stem cell in LIF-supplemented culture medium (19, 20, 27-29). 

Moreover, LIF is required for the lesion-induced proliferation of neuronal progenitors in the regenerating adult olfactory suggesting that LIF may promote neurogenesis after other types of injury (28, 30).

LIF acts through the LIF cell surface receptor complex (9). This factor activates three signaling intracellular pathways including the JAK/ STAT3, MAPK2/SHP and PI/AKT3K upon binding to the LIFr complex (9, 31, 32). These pathways regulate the gene expression pattern in target cells and play a crucial role in activation of LIF that promotes the proliferation of stem cells (32). Moreover, LIF, as an anti-apoptotic factor, induces proliferation of human endometrial stem cells by avoiding the apoptosis (13, 14, 29). In the present study, CD90 expression increased in human endometrial stem cells treated with LIF. This result showed that LIF had a positive effect on the population of mesenchymal stem cells.

In another point of the present study by quantitative analysis, Oct4 and Nanog genes expression, which were recognized as pluripotency markers, enhanced in human endometrial stem cells in treatment with LIF. This result indicated that increasing of Nanog gene expression in human endometrial stem cells could be related to maintenance of undifferentiating and pluripotency. According to our results, it is indicated that expression of Oct4 is necessary for the maintenance of pluripotency, self-renewal, and proliferation of stem cells (33). Self-renewal process of stem cells is dependent on external and internal signaling. External signaling like LIF can maintenance stem cell pluripotency through activation of nuclear reprogramming factor STAT3. In the other hand, Oct4, Sox2, and Nanog as a central network play a critical role in regulation self-renewal of embryonic stem cells. These three pluripotency genes involved in regulation of other genes and together at the same time perform as an activator of other genes related to self-renewal of stem cells (34).

Similar to our results, Chieregato *et al* reported that mesenchymal stem cells derived from fatty cells treated with LIF expressed PCNA at a high level (35). Hu *et al* showed that the LIF in combination with bFGF up-regulated the expression of pluripotency genes including PCNA, OCT4, and NANOG in human umbilical cord mesenchymal stem cells (15). PCNA, as criteria, has been used for evaluation of cell proliferation (35). PCNA exists in the nucleus of proliferating cells, therefore its expression increases in human endometrial stem cells treated with LIF compared to control groups.

It is still not obvious how LIF affect proliferation of mesenchymal cells, but our observations support the hypothesis that proliferation of stem cells may be due to linking LIF to its receptor (LIFr) on the surface of the cells. Endogenous LIFr signaling promotes the maintenance of embryonic and adult stem cells in vivo and in vitro (36, 37). It is suggested that addition of LIF to the culture medium exogenously increases expression of LIFr. Therefore increasing in the expression of LIFr in stem cells treated with LIF can be due to positive feedback between LIF and LIFr. Also a high level of LIFr expression cause increase in its production.

## Conclusion

In conclusion, the present study suggests that LIF has a great impact on proliferation, survival, and maintenance of pluripotency of human endometrial stromal cells and it could be applicable in cell therapies.

## References

[1] Mutlu L, Hufnagel D, Taylor HS (2015). The Endometrium as a Source of Mesenchymal Stem Cells for Regenerative Medicine. Biol Reprod.

[2] Chan RW, Schwab KE, Gargett CE (2004). Clonogenicity of human endometrial epithelial and stromal cells. Biol Reprod.

[3] Fayazi M, Salehnia M, Ziaei S (2014). Differentiation of human CD146-positive endometrial stem cells to adipogenic-, osteogenic-, neural progenitor-, and glial-like cells. In Vitro Cell Dev Biol Anim.

[4] Fayazi M, Salehnia M, Ziae S Characteristics of Human Endometrial Stem Cells in Tissue and Isolated Cultured Cells: An Immunohistochemical Aspect. Iran Biomed J.

[5] Schwab K, Gargett C (2007). Co-expression of two perivascular cell markers isolates mesenchymal stem-like cells from human endometrium. Hum Reprod.

[6] Wolff EF, Wolff AB, Du H, Taylor HS (2007). Demonstration of multipotent stem cells in the adult human endometrium by in vitro chondrogenesis. Reprod Sci.

[7] Schwab K, Hutchinson P, Gargett C (2008). Identification of surface markers for prospective isolation of human endometrial stromal colony-forming cells. Hum Reprod.

[8] Dominici M, Le Blanc K, Mueller I, Slaper-Cortenbach I, Marini F, Krause D (2006). Minimal criteria for defining multipotent mesenchymal stromal cells The International Society for Cellular Therapy position statement. Cytotherapy.

[9] Nicola NA, Babon JJ (2015). Leukemia inhibitory factor (LIF). Cytokine Growth Factor Rev.

[10] Nishishita N, Ijiri H, Takenaka C, Kobayashi K, Goto K, Kotani E (2011). The use of leukemia inhibitory factor immobilized on virus-derived polyhedra to support the proliferation of mouse embryonic and induced pluripotent stem cells. Biomaterials.

[11] Ohtsuka S, Nakai-Futatsugi Y, Niwa H (2015). LIF signal in mouse embryonic stem cells. JAKSTAT.

[12] Hirano T, Nakajima K, Hibi M (1997). Signaling mechanisms through gp130: a model of the cytokine system. Cytokine Growth Factor Rev.

[13] Niwa H, Burdon T, Chambers I, Smith A (1998). Self-renewal of pluripotent embryonic stem cells is mediated via activation of STAT3. Genes Dev.

[14] Morton SD, Cadamuro M, Brivio S, Vismara M, Stecca T, Massani M, Bassi N, Furlanetto A, Joplin RE, Floreani A, Fabris L, Strazzabosco M (2015). Leukemia inhibitory factor protects cholangiocarcinoma cells from drug-induced apoptosis via a PI3K/AKT-dependent Mcl-1 activation. Oncotarget.

[15] Hu WL, Wu PP, Yin CC, Shi JM, Yin M (2016). Effects of Leukemia Inhibitory Factor Combined with Basic Fibroblast Growth Factor on Self-maintenance and Self-renewal of Human Umbilical Cord Mesenchymal Stem Cells In Vitro. Zhongguo Shi Yan Xue Ye Xue Za Zhi.

[16] Morgani SM, Brickman JM (2015). LIF supports primitive endoderm expansion during pre-implantation development. Development.

[17] Niwa H, Miyazaki J-i, Smith AG (2000). Quantitative expression of Oct-3/4 defines differentiation, dedifferentiation or self-renewal of ES cells. Nature Gen.

[18] Lo NW, Intawicha P, Chiu YT, Lee KH, Lu HC, Chen CH (2015). Leukemia inhibitory factor and fibroblast growth factor 2 critically and mutually sustain pluripotency of rabbit embryonic stem cells. Cell Transplant.

[19] Wang P, Suo LJ, Wang YF, Shang H, Li GX, Hu JH, Li QW (2014). Effects of GDNF and LIF on mouse spermatogonial stem cells proliferation in vitro. Cytotechnology.

[20] Mirzapour T, Movahedin M, Tengku Ibrahim TA, Koruji M, Haron AW, Nowroozi MR, Rafieian SH (2012). Effects of basic fibroblast growth factor and leukaemia inhibitory factor on proliferation and short-term culture of human spermatogonial stem cells. Andrologia.

[21] Hirata H, Kawamata S, Murakami Y, Inoue K, Nagahashi A, Tosaka M (2007). Coexpression of platelet-derived growth factor receptor alpha and fetal liver kinase 1 enhances cardiogenic potential in embryonic stem cell differentiation in vitro. J Biosci Bioeng.

[22] Kao A-P, Wang K-H, Chang C-C, Lee J-N, Long C-Y, Chen H-S (2011). Comparative study of human eutopic and ectopic endometrial mesenchymal stem cells and the development of an in vivo endometriotic invasion model. Fertil Steril.

[23] Alberts B, Johnson A, Lewis J (2002). Molecular Biology of the Cell.

[24] Singh VK, Fatanmi OO, Singh PK, Whitnall MH (2012). Role of radiation-induced granulocyte colony-stimulating factor in recovery from whole body gamma-irradiation. Cytokine.

[25] Pfaffl MW (2001). A new mathematical model for relative quantification in real-time RT–PCR. Nucleic Acids Res.

[26] Carter DA, Dick AD, Mayer EJ (2009). CD133+ adult human retinal cells remain undifferentiated in Leukaemia Inhibitory Factor (LIF). BMC Ophthalmol.

[27] Nikolova DB, Martinova YS, Seidensticker M, Bellvé AR (1996). Leukaemia inhibitory factor stimulates proliferation of prospermatogonial stem cells. Reprod Fertil Dev.

[28] Bauer S, Patterson PH (2006). Leukemia inhibitory factor promotes neural stem cell self-renewal in the adult brain. J Neurosci.

[29] Furue M, Okamoto T, Hayashi Y, Okochi H, Fujimoto M, Myoishi Y (2005). Leukemia inhibitory factor as an anti-apoptotic mitogen for pluripotent mouse embryonic stem cells in a serum-free medium without feeder cells. In Vitro Cell Dev Biol Anim.

[30] Bauer S, Rasika S, Han J, Mauduit C, Raccurt M, Morel G (2003). Leukemia inhibitory factor is a key signal for injury-induced neurogenesis in the adult mouse olfactory epithelium. J Neurosci.

[31] Chang M, Park C, Son H, Lee Y, Lee S (2004). Developmental stage-dependent self-regulation of embryonic cortical precursor cell survival and differentiation by leukemia inhibitory factor. Cell Death Differ.

[32] He F, Ge W, Martinowich K, Becker-Catania S, Coskun V, Zhu W (2005). A positive autoregulatory loop of Jak-STAT signaling controls the onset of astrogliogenesis. Nat Neurosci.

[33] Matthai C, Horvat R, Noe M, Nagele F, Radjabi A, Van Trotsenburg M (2006). Oct-4 expression in human endometrium. Mol Hum Reprod.

[34] Loh Y-H, Wu Q, Chew J-L, Vega VB, Zhang W, Chen X (2006). The Oct4 and Nanog transcription network regulates pluripotency in mouse embryonic stem cells. Nat Genet.

[35] Chieregato K, Castegnaro S, Madeo D, Astori G, Pegoraro M, Rodeghiero F (2011). Epidermal growth factor, basic fibroblast growth factor and platelet-derived growth factor-bb can substitute for fetal bovine serum and compete with human platelet-rich plasma in the ex vivo expansion of mesenchymal stromal cells derived from adipose tissue. Cytotherapy.

[36] Pitman M, Emery B, Binder M, Wang S, Butzkueven H, Kilpatrick T (2004). LIF receptor signaling modulates neural stem cell renewal. Mol Cell Neurosci.

[37] Shimazaki T, Shingo T, Weiss S (2001). The ciliary neurotrophic factor/leukemia inhibitory factor/gp130 receptor complex operates in the maintenance of mammalian forebrain neural stem cells. J Neurosci.

